# Computational design of improved standardized chemotherapy protocols for grade II oligodendrogliomas

**DOI:** 10.1371/journal.pcbi.1006778

**Published:** 2019-07-15

**Authors:** Víctor M. Pérez-García, Luis E. Ayala-Hernández, Juan Belmonte-Beitia, Philippe Schucht, Michael Murek, Andreas Raabe, Juan Sepúlveda

**Affiliations:** 1 Department of Mathematics, Mathematical Oncology Laboratory (MOLAB), Universidad de Castilla-La Mancha, Avda. Camilo José Cela, 3, 13071 Ciudad Real, Spain; 2 Departamento de Ciencias Exactas y Tecnología Centro Universitario de los Lagos, Universidad de Guadalajara, Lagos de Moreno, Mexico; 3 Universitätsklinik für Neurochirurgie, Bern University Hospital, CH-3010 Bern, Switzerland; 4 Oncology Unit, Hospital 12 de Octubre, Avda. de Córdoba s/n, 28041 Madrid, Spain; University at Buffalo - The State University of New York, UNITED STATES

## Abstract

Here we put forward a mathematical model describing the response of low-grade (WHO grade II) oligodendrogliomas (LGO) to temozolomide (TMZ). The model describes the longitudinal volumetric dynamics of tumor response to TMZ of a cohort of 11 LGO patients treated with TMZ. After finding patient-specific parameters, different therapeutic strategies were tried computationally on the ‘in-silico twins’ of those patients. Chemotherapy schedules with larger-than-standard rest periods between consecutive cycles had either the same or better long-term efficacy than the standard 28-day cycles. The results were confirmed in a large trial of 2000 virtual patients. These long-cycle schemes would also have reduced toxicity and defer the appearance of resistances. On the basis of those results, a combination scheme consisting of five induction TMZ cycles given monthly plus 12 maintenance cycles given every three months was found to provide substantial survival benefits for the in-silico twins of the 11 LGO patients (median 5.69 years, range: 0.67 to 68.45 years) and in a large virtual trial including 2000 patients. We used 220 sets of experiments in-silico to show that a clinical trial incorporating 100 patients per arm (standard intensive treatment versus 5 + 12 scheme) could demonstrate the superiority of the novel scheme after a follow-up period of 10 years. Thus, the proposed treatment plan could be the basis for a standardized TMZ treatment for LGO patients with survival benefits.

## Author summary

We developed a mathematical model describing the longitudinal volumetric growth data of grade II oligodendroglioma patients and their response to temozolomide. The model was used to explore alternative therapeutic protocols for the in-silico twins of the patients and in virtual clinical trials. The simulations show that enlarging the time interval between chemotherapy cycles would maintain the therapeutic efficacy, while limiting toxicity and deferring the development of resistance. This may allow for improved drug-exposure by administering a larger number of cycles for longer treatment periods. A scheme based on this idea consisting of an induction phase (5 consecutive cycles, 1 per month) and a maintenance phase (12 cycles given in three-months intervals) led to substantial survival benefits in-silico. The computational results suggest that a clinical trial enrolling 100 patients per arm (standard intensive therapy versus 5+12 novel scheme) could prove the effectiveness of the proposed approach after a follow-up period of 10 years.

## Introduction

Oligodendrogliomas are low-incidence glial tumors, affecting mostly young adults. They are slowly growing, infiltrative tumors with isocitrate dehydrogenase 1 or 2 mutations and codeletion of chromosomal arms 1p and 19q. Grade II oligodendrogliomas (LGOs) are well differentiated tumors with a low mitotic index [1]. In spite of the long median patient survival, they are incurable currently [2].

Many oligodendroglioma patients present few neurological symptoms for extended periods of time. The decision on the specific combination of therapies to be used on each patient is based on the qualitative consideration of different variables including age, tumor grade, performance status and tumor location [3]. Radiation therapy (RT) is beneficial for patients in terms of survival, but its timing has been the subject of debate [4]. Regarding chemotherapy, temozolomide (TMZ), an oral alkylating agent, has a favourable toxicity profile [5] and can contribute to reduction in seizure frequency in low-grade glioma patients [6]. Phase II trials have demonstrated its effectivity against low grade gliomas [7–9]. Also, neoadjuvant chemotherapy given to surgically unresectable tumors has allowed subsequent gross total resection in some cases [10], which is of relevance when the tumour is highly infiltrative or located in eloquent areas. Thus, prolonged TMZ treatment is a relevant option either as up-front or as adjuvant treatment.

Clinical trials have shown a similar efficacy of TMZ vs RT for 1p/19q-codeleted tumors [11, 12]. Also, RT is associated with late neurocognitive toxicity. Thus chemotherapy is frequently used as first-line treatment for oligodendroglioma patients. In that context, relevant questions arise such choice of the chemotherapy regimen and the optimal number of cycles to be prescribed.

Mathematical models have potential to help in finding optimized treatment schedules/combinations improving survival and/or reducing toxicity [13, 14]. Once the base mathematical model is set, patient-specific parameters can be obtained from data. That provides an ‘in-silico twin’ [15] allowing computational studies that could be beneficial for real patients.

## Materials and methods

### Ethics statement

The study was approved by Kantonale Ethikkommission Bern (Bern, Switzerland), with approval number: 07.09.72.

### Patients

82 patients diagnosed with low-grade gliomas (biopsy/surgery confirmed astrocytoma, oligoastrocytoma or oligodendroglioma according to the WHO 2007 classification) and followed at the Bern University Hospital between 1990 and 2013 were initially included in the study.

Of that patient population, we selected grade II 1p/19q-codeleted tumors (thus LGOs according to the 2016 WHO classification) 36 patients. 17 of those patients did not receive TMZ and 2 received TMZ in combination with other therapy. Finally two of them received TMZ only after becoming anaplastic tumors. Of the remaining 16 patients, three did not respond to TMZ and two responded initially but progressed during treatment to anaplastic forms. Thus 11 oligodendroglioma patients treated with at least three cycles of TMZ, responded to the therapy, had neither previous RT treatment nor other treatment given in the period of study and did not display any signs of malignant transformation.

### Image acquisition and analysis

Radiological glioma growth was quantified by manual measurements of tumour diameters on successive MRIs (T2/FLAIR sequences). Since some of the older patients were available only as jpeg images we computed the volume using the ellipsoidal approximation. The three largest tumour diameters (*D*_1_, *D*_2_, *D*_3_) along the axial, coronal and sagittal planes were measured and tumour volumes estimated using the equation *V* = (*D*_1_ ⋅ *D*_2_ ⋅ *D*_3_)/2, following the standard practice [16]. To estimate the error of the methodology we took a different set of glioma patients from another study [17] and compared their volumes computed accurately using a semi-automatic segmentation approach with those computed using the ellipsoidal approximation. Mean differences were 18%, that was the reference level used for the error in the volume computations.

### Mathematical model

In this paper we considered LGOs in a simplified way as composed of two tumor cell compartments. The first one was the tumor cell population *P*(*t*), assumed to grow logistically. The second one was lethally damaged tumor cells because of the action of the therapy *D*(*t*). Temozolomide effect on proliferative cells is a complex one, leading to death through different ‘programmes’ [18–20]. We put together the different processes into two groups, each described by a term in our equations. The first one was early death accounting for necrosis, autophagy and drug-induced apoptosis with rate *α*_1_. The second one was delayed death through mitotic catastrophe with rate *α*_2_. The drug concentration in tissue was described by the function *C*(*t*) with an elimination rate constant, λ.

**Fig 1:**
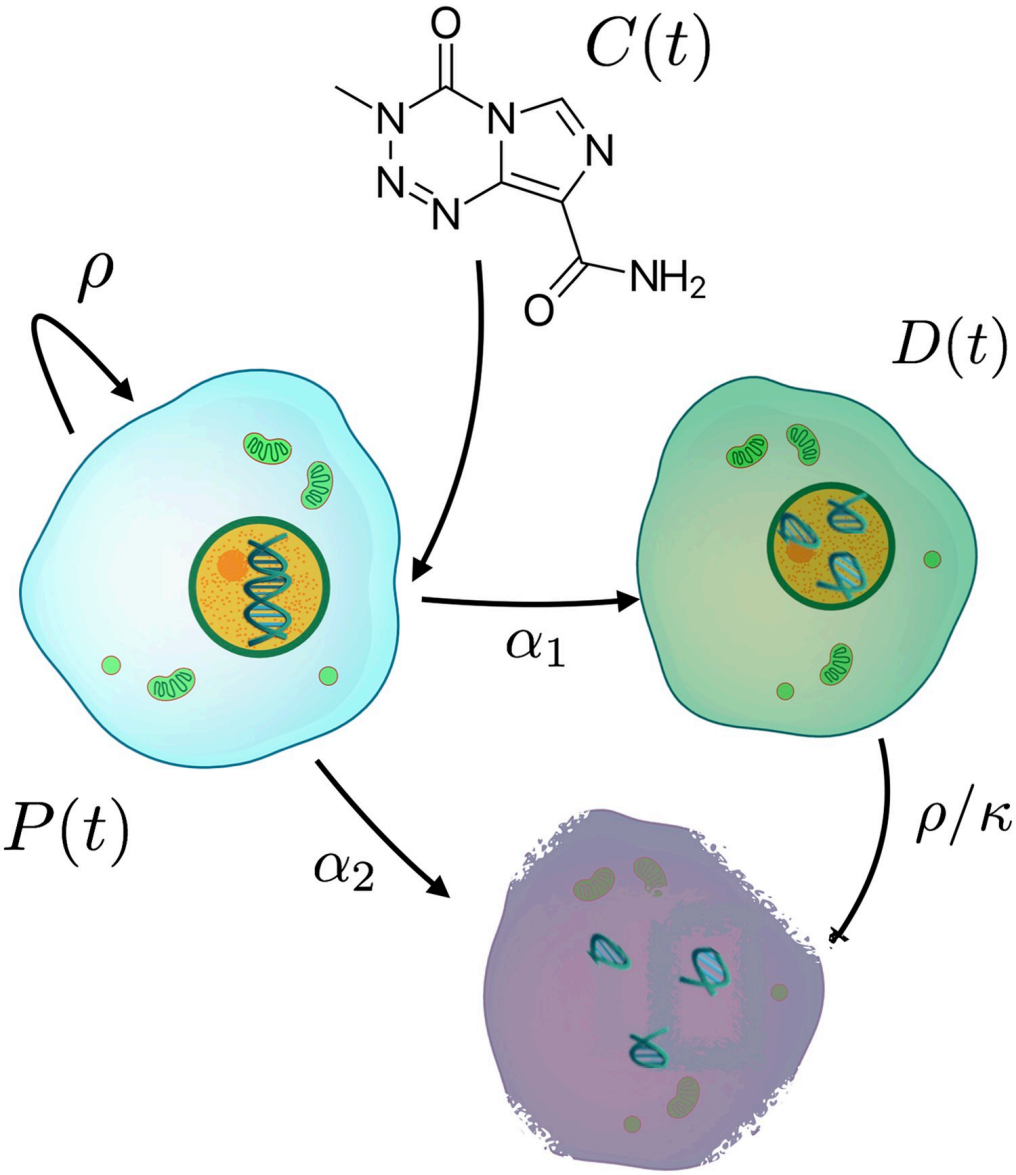
Schematic description of the model defined by Eq (1). Tumor cell population *P*(*t*) grows at a rate *ρ* and saturates at a maximum size *K*. These cells are killed by the drug *C*(*t*) (and removed) through direct kill mechanisms *α*_2_*P*(*t*)*C*(*t*). A number of cells *α*_1_*P*(*t*)*C*(*t*) moves into a different compartment of lethally damaged cells *D*(*t*). Damaged cells die at a rate *ρ*/*κ*[1 − (*P* + *D*)/*K*] because of the mitotic catastrophe.

Fig 1 shows a schematic description of the model. The equations were:

$$\frac{dP}{dt}=\rho P(1-\frac{P+D}{K})-\alpha_{1}PC-\alpha_{2}PC,$$ (1a)

$$\frac{dD}{dt}=-\frac{\rho }{\kappa }D(1-\frac{P+D}{K})+\alpha_{1}PC,$$ (1b)

$$\frac{dC}{dt}=-\lambda C,$$ (1c)

Chemotherapy was described by a sequence of doses *d* given at times *t*_1_ < *t*_2_ < … < *t*_*N*_. The initial time corresponding to the first volumetric observation was denoted as *t*_0_. Initial conditions for Eq (1) were taken to be *P*_0_ = *P*(*t*_0_), *D*(*t*_0_) = *C*(*t*_0_) = 0. Let us define $f\left(t_{j}^{-}\right)={\text{lim}}_{t\to t_{j}^{-}}f\left(t\right)$ and *C* is the fraction of the dose *d* reaching brain tumor tissue, assumed to to be estimated later. Drug administration was described as impulses for the times *t*_*j*_ so that $P\left(t_{j}\right)=P\left(t_{j}^{-}\right)$, $D\left(t_{j}\right)=D\left(t_{j}^{-}\right)$, $C\left(t_{j}\right)=C\left(t_{j}^{-}\right)+C$.

Since cell numbers are expected to be proportional to the volumes occupied by each tumor subpopulation we worked with the later quantities, that are directly measurable. Thus, *P*(*t*) and *D*(*t*) through this paper were measured in cm^3^.

### Parameter estimation

We chose the parameter *κ*, corresponding to the averaged number of cell divisions before death by mitotic catastrophe to be equal to 1. In mitotic catastrophe, damaged cells do not die until they try to perform mitosis again. Although a few cells may be able to complete more than one cycle, most of them will die in the first one [21]. This is a reasonable assumption allowing us to get rid of this parameter.

The carrying capacity parameter *K* is the one with a less defined value but could be expected to be in a range between 300 and 550 cm^3^. The later number is in line with the maximal volumes observed in low-grade glioma patients [22]. However, many patients die when the tumor volume is smaller [15].

The most typical chemotherapy schedule consists of cycles of 28 days with five TMZ oral doses on days 1 to 5 and then a rest period of 23 days. Typical dose per day is *d* = 150 mg per m^2^ of patient body surface. To calculate the rate of drug decay λ we followed the same methodology as in Ref. [26], using values of TMZ half-life clearance time *t*_1/2_ for doses of 150 mg/m^2^. From the definition of *t*_1/2_ and since Eq (1c) has exponentially decaying solutions we obtain that 1/2 = exp(−λ*t*_1/2_). To account also for the drug loss during transport to the brain we computed the value of the dose getting to the tumour as *C* = *β* ⋅ *d* ⋅ *b*, where *β* is the fraction of TMZ getting to 1 cm^3^ of brain interstitial fluid (from a unit dose) and *b* is the patient’s body surface. Then *C*_0_ can be interpreted as an effective dose per fraction. The parameter *β* can be calculated using the value of maximal TMZ concentration *C*_max_ for a dose of 150 mg/m^2^ taken from the literature [23]. Since time to reach peak drug concentration in brain is smaller than two hours and thus negligible in comparison with the other time scales in the model, we chose to set the initial drug concentration *C*_0_ to the value *C*_max_ = 0.6 *μ*g/cm^3^ as in Ref. [26].

The parameters *α*_1_, *α*_2_ and *ρ* are expected to depend strongly on the tumor growth rate and sensitity to the therapy and will be considered to be adjustable parameters. These parameters, together with the initial population value *P*(0) were fit for each patient’s longitudinal volumetric data using the library fmincon in the scientific software package Matlab (R2017b, The MathWorks, Inc., Natick, MA, USA). Table 1 summarizes the main characteristics and parameter values found for patients included in the study. The fits obtained with those parameters were accurate, with a low relative error as computed as the difference between the tumor volume data and those obtained by the best fit. Mathematically $\bar{e}=\left(100/N\right)\sum_{i=1}^{N}\left|V_{ip}-V_{if}\right|/V_{ip}$ (expressed in percent), where *V*_*ip*_ was tumour volume data and *V*_*if*_ was the tumour volume calculated with the parameters obtained by the least square fitting. The average of the relative errors for all the simulations was 9.1% and the median 8.3%, that were within the volume measurement uncertainty.

**Table 1:** Parameter values best describing the longitudinal volumetric data for the patients included in the study. Values for the other parameters were fixed to *K* = 523.6 cm^3^, λ = 8.3184 day^−1^.

ID	# TMZ cycles	*P*(0)	*ρ* (day^−1^)	*α*_1_ cm^3^/(*μ*g day)	*α*_2_ cm^3^/(*μ*g day)
6	11	46.0	1.01 × 10^−3^	0.32	0.1
10	15	45.4	7.06 × 10^−4^	0.27	0.21
25	4	31.7	1.84 × 10^−3^	0.76	0.75
57	9	46.8	8.74 × 10^−4^	0.23	0.21
105	20	145.0	2.05 × 10^−3^	0.1	0.14
108	5	13.9	1.73 × 10^−3^	0.92	0.18
151	11	34.6	7.41 × 10^−4^	0.52	0.05
159	11	64.7	5.33 × 10^−4^	0.57	0.6
170	17	6.4	2.28 × 10^−3^	0.01	0.28
203	9	29.8	3.31 × 10^−4^	1.99	0.1
213	18	23.9	6.08 × 10^−4^	0.3	0.3

Numerical simulations of Eq (1) were performed using the Matlab library ode45. Results for the parameters are listed in Table 1.

### Comparison of different drug schedules in-silico

To test the effect of different drug schedules for each virtual patient obtained from our data we performed series of numerical simulations.

A first set of simulations consisted of a schedule were five daily doses of the drug were given on days 1-5 of the cycle and then the standard waiting period of 23 days was increased with variable times of up to 6 months with intervals of 15 days, i.e. 12 different spacings were studied for each virtual patient. These strategies will be denoted to as ‘long-cycle’ ones in what follows.

Other sets of simulations consisted on the redistribution of the five doses during the 28 days of the cycle in two different ways, to be denoted hereafter as ‘distributed dose’ regimens. A first distributed regime consisted in 5 doses given following a 1-day on, 1-day off scheme during the first 10 days of the cycle. A second alternative was distributing doses evenly within the cycle duration, i.e. giving a single dose every 4 days.

### Virtual clinical trials

To study the effect of the different treatment schedules on patient survival we designed virtual trials. A number of virtual patients was generated by a random choice of the parameters. Uniform distributions were taken for the parameters in the most representative region of the parameter space obtained from Table 1: *ρ* ∈ [0.5 × 10^−3^, 2.5 × 10^−3^] day^- 1^, *α*_1_ ∈ [0.01, 1.0] cm^3^/*μ*g day, *α*_2_ ∈ [0.1, 0.75] cm^3^/*μ*g day, *P*(0) ∈ [20, 200], *K* ∈ [300, 550] cm^3^. Parameters were randomly sampled from the distributions in order to perform the simulations. 2000 patients were included in each virtual trial (1000 patients on each of the two arms considered). Virtual trials were run using Matlab 2017b parallel computing toolbox using a parallel algorithm on a 64 GB memory 2.7 GHz 12-core Mac pro workstation under OS X 10.14.

For the survival studies patients were assumed to die when tumors reached a volume of 280 cm^3^ and those alive after 25 years were considered as censored events. That volume would correspond to a sphere of 8 cm in diameter. Previous theoretical studies of glioblastoma growth have found the fatal tumor burden to be around 7 cm in diameter [15]. Since low-grade gliomas are less aggressive and harmful for the brain we chose the lethal size to be somewhat larger. In real life that number would obviously depend on tumor location, aggressiveness, patient overall status, etc.

## Results

### The mathematical model describes the response of LGOs to temozolomide

**Fig 2:**
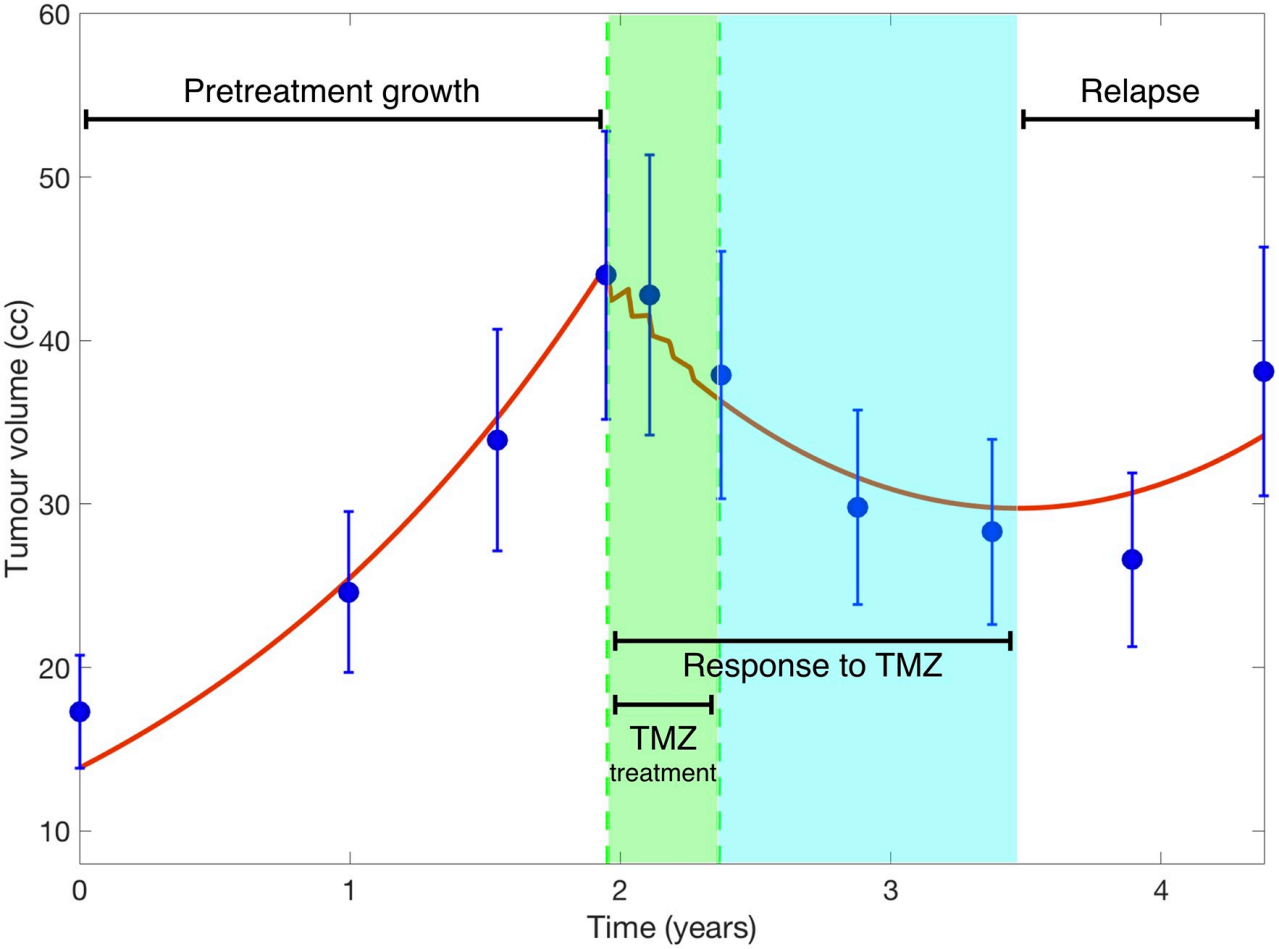
Tumor volumetric longitudinal evolution (solid blue circles) of a patient receiving five chemotherapy cycles together with the best fit found using Eq (1) (red lines). Several stages are observed: Pretreatment growth, response during and after TMZ treatment and tumor relapse. Treatment time is highlighted with a green background and the period of time with response to TMZ without treatment with a light blue background.

Typical low-grade glioma longitudinal growth and response to therapy consists of four stages (see Fig 2). First, without treatment tumor grows slowly but steadily [24]. Next, there is an early ‘fast’ tumor volume reduction associated to the start of treatment with TMZ. Finally, after treatment cesation, there is a long-term response. For the patient shown in Fig 2, the tumor volume reduction lasted for 14 months after the end of the treatment course. Finally, the tumor regrew leading to a clinical relapse. All of those stages were described by the mathematical model. Each stage was associated to one of the biological phenomena reflected as terms in the model equations.

**Fig 3:**
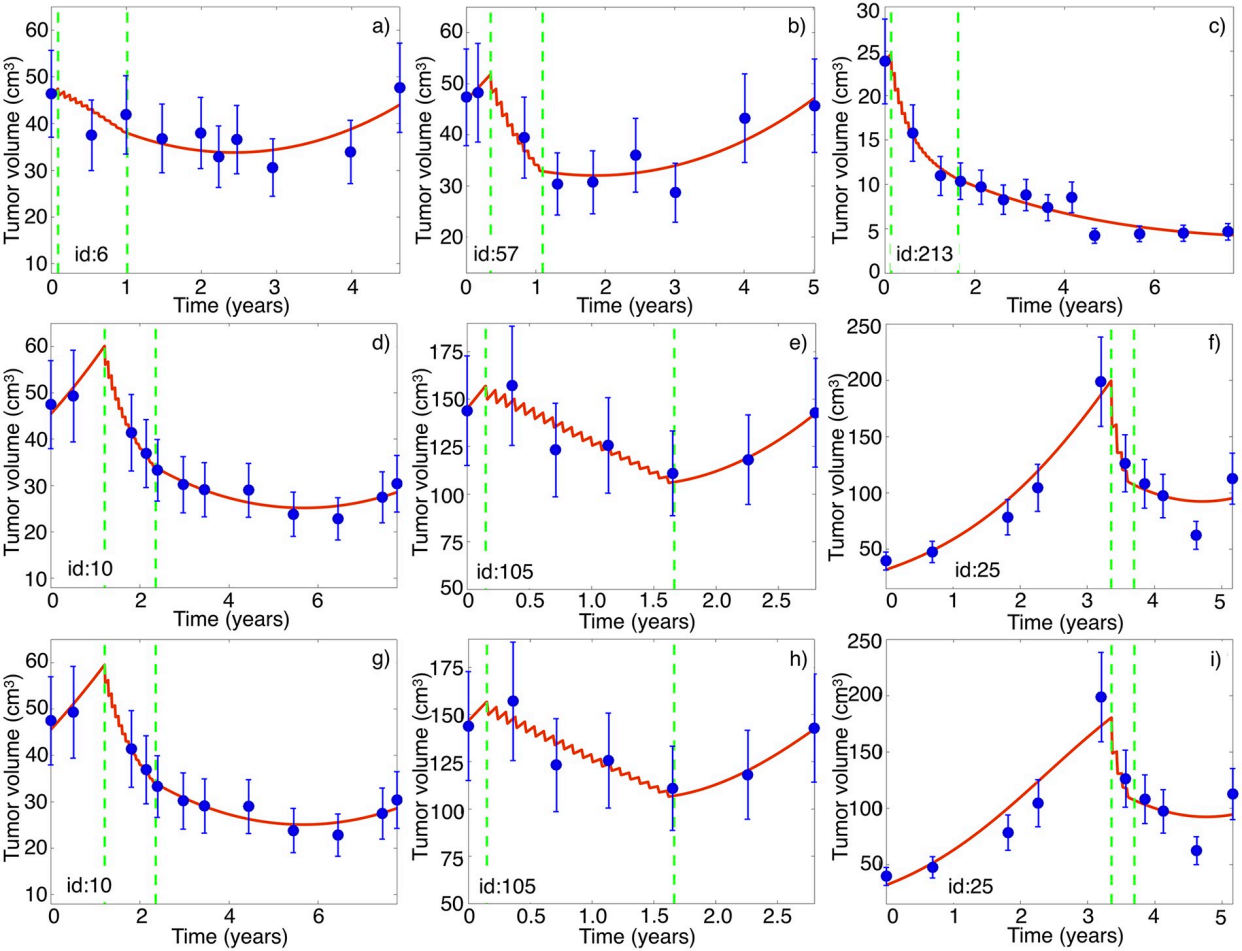
Longitudinal volumetric tumor data (blue circles) and best fits obtained with the model given by Eq (1) (red lines). (a-f) Results for six randomly chosen patients from our dataset for a carrying capacity K = 523.6 cm^3^. (g-i) Results for three patients for K = 261.8 cm^3^. The vertical dashed lines in each subplots mark the start and end times of treatment with TMZ. Patient id’s are indicated in the lower left part of the subplots.

We studied the ability of our mathematical model to describe the tumor responses to TMZ. To do so, we fitted the parameters in Eq (1) using the longitudinal volumetric data for each patient in our cohort. Fig 3 shows results for selected patients. The model described the longitudinal tumor volumetric data in all cases. This validates the choice of biological mechanisms used in constructing the model.

Results shown in Fig 3 were obtained for a fixed (i.e. non fitted) value of the carrying capacity *K* = 523.6 cm^3^. This parameter provides an estimate of the tumor size for which geometrical and other constraints have a substantial influence on the tumor growth rate. Specifically, this is the volume of a sphere of radius 5 cm, a number that is about 2/5 of the whole brain volume [25]. A tumor of this size would be incompatible with the patient’s life and very difficult to obtain given the brain physical barriers. This is the *K* value that will be taken for most of the paper simulations.

However, similar results were obtained for a broad range of values of *K*. As an example, Fig 3(g)–3(i) shows results for selected patients using a smaller *K* = 261.8 cm^3^. Indeed, the value of *K* cannot be uniquely determined from the data. Different values of *K* lead to slightly different fitting parameter choices and similar shapes of the fitting curves. Thus we chose to fix it through the paper. However the results to be presented do not depend on the specific choice of *K*.

### Simulations show potential benefits of alternative treatment schedules

**Fig 4:**
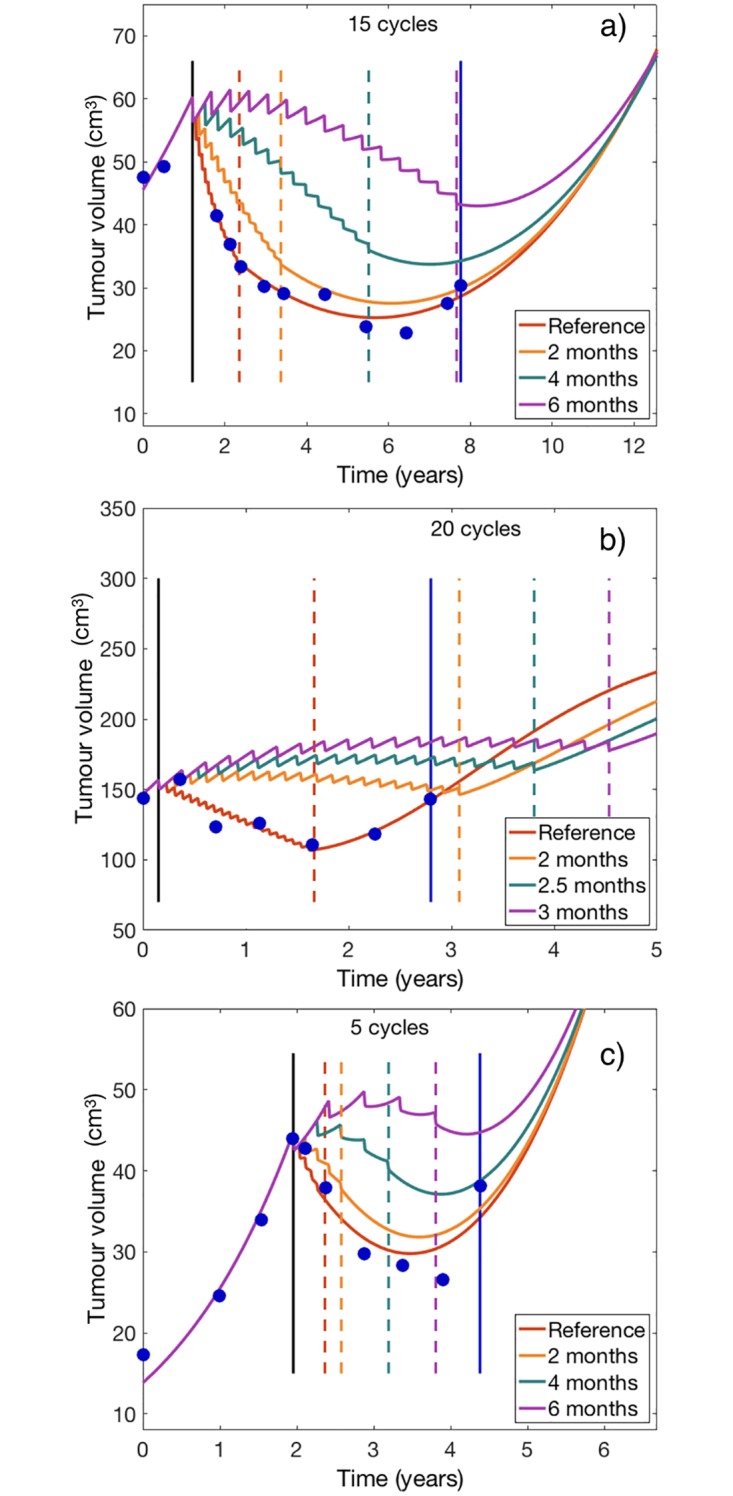
Simulated tumor growth curves under alternative treatment schedules with variable time spacing between consecutive cycles. Eq (1) were solved for each patient with the best fitted parameters under the different treatment treatment regimens. Each subplot shows the reference (fitted to data) growth curve in red and the simulated growth curves under three alternative schemes with spacing between doses of: (a, c) 2, 4, and 6 months, and (b) 2, 2.5 and 3 months. Vertical dashed lines indicate the end times of the different treatment regimens. The vertical solid blue lines mark the time domain for which imaging follow-up data was available for the patient.

The model was then used as a discovery platform to test alternative treatment regimens in-silico for the patients included in the study. As a first test, we enlarged the time interval between cycles. Five daily doses of the drug were given on days 1-5 of the cycle and then the standard waiting period of 23 days was increased as described in methods. In general, the long-term tumor evolution was similar for all the schedules when the cycle’s length was in the range 1-4 months. Thus, from the volumetric point of view, all schedules led to similar asymptotic dynamics for the virtual patients. Results for selected patients are shown in Fig 4. Long-cycle treatment regimens resulted in smaller tumor volume reduction due to the less intensive nature of the schemes.

For small tumors, the nonlinear terms in Eq (1) played a marginal role as far as the values of (*P* + *D*)/*K* were small. However, in the case of large tumors, whose *P* + *D* size was comparable to *K*, the nonlinear terms had a substantial contribution to the dynamics ruled by Eq (1). In the latter case, differences between the schemes were observed favoring long-cycle schemes (see e.g. Fig 4(b)).

As a second series of tests, we explored alternative treatment regimens based on the 28-day cycle, the distributed dose regimens as described in ‘Methods’. All distributed treatment regimens led to tumor volumetric evolutions equivalent to the ones of the standard treatment (e.g., those depicted in Fig 3).

Several virtual trials were conducted as described in the ‘Methods’ section. Benefits in median survival were found for the long-cycle strategies that were dependent on the parameter *K*. Long-cycle treatment schemes were never inferior in terms of survival to the standard ones. Indeed, the differences found between survival curves for long-cycle schedules versus the standard ones were never statistically significant (*p* < 0.05) according to the log-rank test.

### A combined treatment regime provided survival advantages in-silico and may provide a standard for LGO patients

Patients in our retrospective dataset were treated with a variable number of TMZ cycles (mean 12, range 4-20, see Table 1). Treatment was effective for all patients included. However, since there is no standardized protocol for chemotherapy in LGO patients, the decision to stop treatment was taken depending on toxicity, physician and patient preferences, etc.

**Fig 5:**
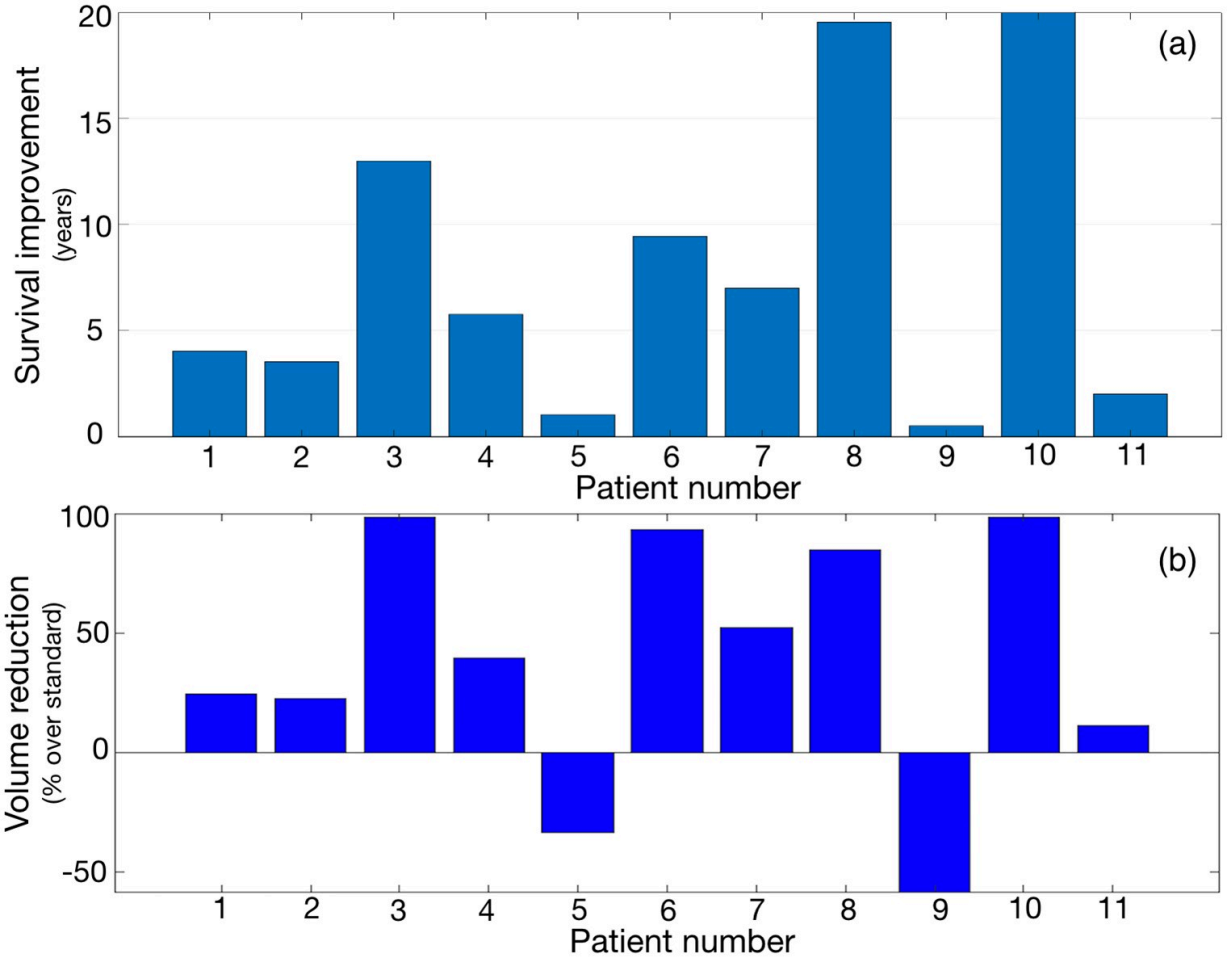
Benefits of the proposed combined treatment with 5 induction cycles given monthly and 12 maintenance cycles given every three months. (a) Predicted survival benefits for the virtual patients subjected to the proposed scheme in comparison with survival of the real patients. (b) Maximum volume reduction obtained by the proposed scheme in comparison with the maximum volume reduction achieved for the real patient.

Next we explored the potential effectiveness of standardizing treatment for the virtual patients obtained from our patient’s data in-silico. To do so we tested our scheme consisting of an induction part of five cycles given monthly to substantially reduce the tumor burden followed by a consolidation of 12 cycles given every three months. This treatment scheme was based on the idea that TMZ cycles given every three months should be well tolerated and allow for this long schedule. Moreover, having a first induction part would result in an initial larger tumor volume reduction than for the long-cycle schemes alone. Results are summarized in Fig 5. Survival improvements, many of them substantial, were obtained for the virtual counterparts of the patients included in the study (Median 5.69 years, range: 0.67 to 68.45 years, see Fig 5(a)). Virtual patients for which the number of cycles was larger (patients 3, 6, 7, 8 and 10) than those received by the real one (see Table 1) had larger survival benefits. Also for most patients there was a substantial volumetric reduction in relation to the one achieved for the real patient under the number of cycles given by Table 1 (see Fig 5(b)).

**Fig 6:**
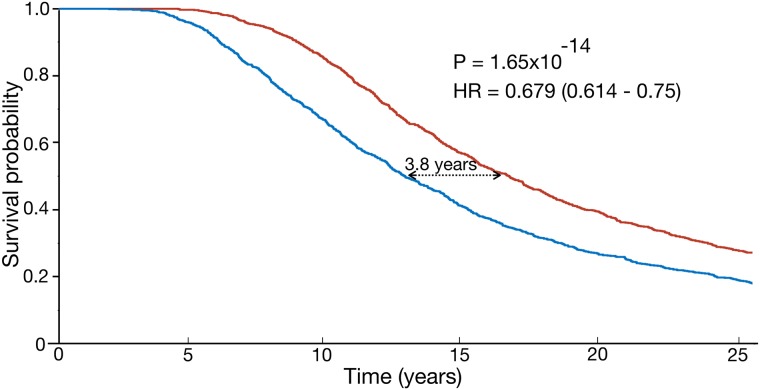
Results of the virtual trial comparing a standard chemotherapeutic approach for LGO versus the proposed scheme. Shownare the Kaplan-Meir plots for both arms. In the first arm (blue), virtual patients received a random number of cycles in the range spanned by the real patients (between 5 and 18 sequential cycles with the standard 1 month spacing). The same virtual patients received the proposed scheme (5 cycles induction given monthly + 12 cycles consolidation given every 3 months).

Although those results are interesting, they hold for a limited number of patients. Thus, to complement the previous results we developed a virtual trial with 2000 virtual patients included in two arms as described in ‘Methods’. Results are summarized in Fig 6. Differences between the curves were statistically significant (*p* = 1.65 × 10^−14^, HR = 0.679 (0.614−0.75)), with a difference in median survival of 3.8 years between both treatment arms.

**Fig 7:**
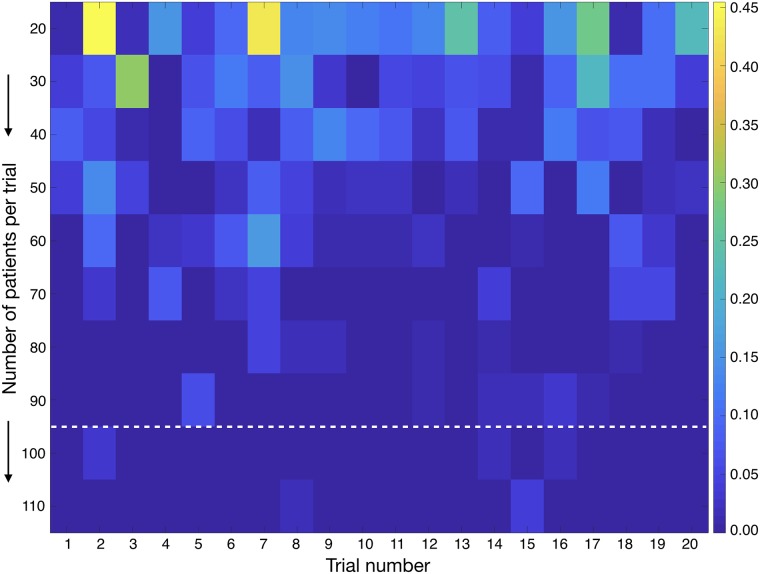
Virtual clinical trials with 100 or more patients per arm (standard intensive versus 5 + 12 scheme) with 10 years followup achieve statistically significant survival differences between different treatment groups. The pseudocolor plot shows the p-value for the difference between both treatment arms for each number of patients *N* and trial out of the 20 performed for each population. In each trial parameters were sampled randomly as described in ‘Methods’. The colorbar on the right provides the scale for the p-values. All trials below the white dashed line (corresponding to *N* > 100) achieved statistical significance *p* < 0.05.

The main interest of our results is the possibility of the proposed scheme to become the basis of a clinical trial that could be beneficial for LGO patients. However, real clinical trials are limited in size (number of patients included) and follow-up time. To test the feasibility of a confirmatory clinical trial we performed a series of numerical experiments, whose results are summarized in Fig 7. For an initial set of 20 patients per arm we compared the standard intensive scheme versus the 5+12 one within a followup period of 10 years and compared the survival of both populations. The experiment was repeated 20 times by resampling randomly the parameter distribution as described in ‘Methods’ and in only 5 of the trials (25%) the differences between treatment arms achieved statistical significance *p* < 0.05. Then the patient population was increased in intervals of 10 patients until a final population of 110 per treatment arm. For each patient population, 20 trials were developed in-silico. As the patient number increased the statistical significance of the trials was better. For the 20 trials performed with 100 patients per arm and 110 patients per arm all p-values were below the statistical significance limit *p* = 0.05 (Fig 7). Thus a trial with more than 100 patients per arm with 10 years followup should be able to show the superiority of the 5+12 scheme over the standard one with a high confidence (>97.5%).

## Discussion

Our mathematical model successfully reproduced the tumor size dynamics of LGO patients treated with TMZ. The observed radiological dynamics cannot be described with mathematical models based on instantaneous response to therapy. Thus, a key ingredient in our model was the combination of two different types of death processes, ones leading to ‘early’ cell death (treatment induced apoptosis and necrosis) and others leading to ‘late’ cell death through mitotic catastrophe. This was incorporated through three adjustable parameters. An additional parameter, the carrying capacity, accounts for the limitation of growth due to geometrical constraints. Other parameters were estimated from biological data.

Many authors have built mathematical models to understand and describe different aspects of the natural history and response to treatments of low-grade gliomas [26–37], some of them focusing on CT. Ribba et al. [29] developed a six-parameter model based on proliferative, quiescent and damaged quiescent compartments. Some biological assumptions in the model were debatable: there was no connection from the quiescent cell compartment to the proliferative cell compartment other than through the damaged quiescent cells, the drug was assumed to affect equally proliferative and quiescent cells, and the drug decay time in brain tissue was fitted to be of the order of several months. This is in striking contrast with the value used here inferred from realistic data of a few hours. Bogdanska et al. [26] used a minimal mathematical model incorporating death only through mitotic catastrophe [34], which forced the parameter *κ* to have values beyond the biologically feasible range. Our approach achieved better quantitative fittings than those in Ref. [34] while having all parameter values in meaningful ranges.

Some previous models have explored different therapeutic regimens for low-grade gliomas [27, 28, 31, 34]. First, in Ref. [34] it was proposed that deferring the complete radiotherapy treatment or splitting the treatment into parts separated by long periods of time would not affect patient survival in a mathematical model of low-grade glioma response with delayed death. Then, in the framework of the same model, Pérez-García and Romasanta [28] showed that protracted radiotherapy schemes with the standard dose of 1.8 Gy per fraction could lead to substantial improvements in survival if the time spacing between doses was maintained below a certain threshold. This result was later extended for different doses per fraction and time spacing between doses in Ref. [27]. Mazocco et al [31] found a similar result using in-silico simulations of the Ribba’s et al model [29].

As to the modeling of the response to the drug we used a linear exposure-response relationship. More sophisticate and realistic models such as *E*_max_ models could have been used. We chose instead the simple linear relationship in line with other previous modeling works [29, 31] because of two reasons. The first one was that more complex and realistic models would imply fixing an additional parameter and making the model more sensitive to overfitting, an effect that we wanted to avoid. Secondly we just fitted the cell damage parameters that best described the response to the dose that what assumed to be always the same. In subsequent simulations we did not changed the dose received but only the spacing between doses. More sophisticate studies considering changing not only the dose interspacing but also the amount of drug given per dose should consider more detailed dose response models.

Here, we used our newly developed model that includes more realistic biological mechanisms to describe the effect of temozolomide on oligodendrogliomas. For all virtual patients the simulations showed interesting features: (i) Tumor growth was found to be asymptotically similar for different treatment schedules. (ii) There were patients for which a survival increase was observed under the alternative treatment regimens. An obvious implication of (i) and (ii) is that the alternative regimens would have no inferior performance in terms of survival. Our virtual clinical trials also supported those findings.

Those ‘long-cycle’ regimens could have other advantages worth considering. The first one is that they could have substantial benefits in terms of toxicity, pharmacoeconomy and also improving the prognosis. In fact, extending the duration of each cycle is a widely used way to treat toxicities caused by cytotoxics. Another possible benefit of those schemes would be improving drug-exposure in LGOs, by administering a larger number of cycles for longer treatment periods.

In real life, dosing schedules are not chosen solely based on predicted optimal results for tumor reduction. The availability of clinicians and the proximity of patients to treatment centers can also play a large role. In our case, treatment is oral and can be taken by patients at home and thus does not require visits to the hospital’s oncology unit. The 3-month periodicity of the consolidation stage of our proposal has the advantage that magnetic resonance scans and visits to the doctor are often planned with that periodicity. Thus patients could have a control blood test, the MRI and the medical revision on the same day. The doctor would then have an opportunity to check the treatment tolerance, the response to treatment and potential relapses, e.g. in the form of anaplastic transtormation. He/she could also remind the patient to continue with the treatment for the next week when appropriate.

The only drawback of long-cycle regimens was a smaller tumor volume reduction due to their less-intensive nature. Although this smaller reduction did not have effects in terms of survival in-silico it would affect symptoms control in real patients. Thus, we designed a mixed treatment scheme consisting of an intensive induction phase of 5 cycles given once per month together with a maintenance stage of 12 cycles given one every three months. This strategy showed an impressive effect on survival. Only for the two patients receiving longer more intensive treatments in real life, the volumetric reductions obtained in-silico were smaller than the ones observed. In spite of that, patients survived longer in the simulations. Indeed, three patients received in real life the same or more chemotherapy cycles than in our proposed scheme, but our possibly less toxic scheme resulted in longer survival in the computer simulations. The results were confirmed in a virtual trial including 2000 patients and comparing both ‘in-silico’ treatment arms. Thus, our proposal incorporates both the suggestions of the new mathematical model with practical real-world aspects to implement for the first time a realistic protocol based partially on the ideas previously suggested in the theoretical literature [28].

Our results for the distributed dose regimes show that the treatment effectiveness does not depend on the precise time of administration of the dose within the cycle. Thus, choosing one or other regime could be done in terms of toxicity reductions or delaying the appearance of resistant clones. An increasing body of evidence suggests that small subpopulations of cancer cells can evade strong selective drug pressure by entering a ‘persister’ state of negligible growth [38]. This drug-tolerant state has been hypothesized to be part of an initial strategy towards eventual acquisition of bona fide drug-resistance. The induction of persisters in glioma cells has been known to be partially reverted by ‘drug wash-out’ suggesting the contribution of epigenetic mechanisms in drug resistance and supporting the possibility of TMZ rechallenge in glioma patients after prior drug exposure [39], provided there is a sufficiently long waiting time between treatments. Recent experimental results support this hypothesis [40]. In our case, the fact that the maintenance phase of the treatment leaves a time spacing of three months within cycles could be optimal not only in terms of survival but also to avoid persisters becoming resistant tumor cells. Thus, an additional benefit of the proposed schedule would be to delay the appearance of resistances. This is a topic to be further investigated in the future.

In our work, we assumed a direct proportionality between tumor cell number and the observable tumor size on T2/FLAIR, and thus only the tumor volume evolution was described and not the whole spatiotemporal tumor cell density as in reaction-diffusion partial-differential equation based mathematical models (see e.g. [28, 32, 33, 36] and references therein). The reason is that we had only data for tumor volumetric evolution and thus the model was written to match the data. Otherwise we would have more degrees of freedom-parameters (such as the diffusion coefficient) without an experimental source to obtain them, what could again lead to overfitting. However, an interesting extension of this work would be to use models where the tumor cell amplitude and size are independent. The inclusion of cell-motility processes as in reaction-diffusion models could provide a computational platform to study the delay of the tumor’s malignant transformation through alternative treatment regimens. Further research is required to relate the signal obtained from diffusion MRI sequences and/or ADC maps with local cellularity values.

We think that both long-cycle regimens and the proposed regimen based on an induction plus a maintenance phase could be beneficial in terms of toxicity and improved drug-exposure. In our study we used a simplified drug concentration model in which only the drug concentration at the site of action was considered. Our choice was based on the fact that we intended to change the dose interspacing and not increasing overall doses. Also, temozolomide lifetime in tissues is very short, of the order of hours, what is much shorter than spacing between cycles and even doses. However, the consideration of more robust pharmacokinetic mathematical models including systemic drug exposure, and toxicity would be a natural continuation of our study.

The main intention of our work was to provide a theoretical foundation for the development of a clinical trial. Because of the limitation of low-grade glioma experimental models, and specifically grade II oligodendroglioma, it is very difficult to test the effectiveness of our long-cycle strategies neither in-vitro nor in animal models. Thus, this is a good example where computational mathematical models must be useful to provide some preclinical evidences. Our in-silico studies with cohorts of virtual patients show that the comparison between patient populations of 100 individuals per arm followed for 10 years should already show statistically significant differences in survival, what may provide an initial estimate for the development of a phase II-III clinical trial.

### Conclusion

We developed a mathematical model of LGOs response to TMZ describing the longitudinal tumor volumetric dynamics. Once fitted for each patient, the model provided a set of parameters describing the behavior of each of the real patients. When subjected to long-cycle treatment regimens the virtual patients showed similar or better performance in terms of survival. In-silico clinical trials confirmed the results for broader parameter regimens. This long-cycle TMZ schedules could prove beneficial for LGO patients in terms of toxicity. We studied ‘in-silico’ a treatment combining an induction phase of 5 consecutive cycles plus a maintenance phase (12 cycles given in three-months intervals). The improved drug-exposure of this scheme led to substantial survival improvements and a good tumor control in-silico. We hope this computational study could provide a theoretical ground for the development of clinical studies and the definition of standardized TMZ treatment protocols for low-grade oligodendroglioma patients with improved survival.
